# Population dynamics of normal and leukaemia stem cells in the haematopoietic stem cell niche show distinct regimes where leukaemia will be controlled

**DOI:** 10.1098/rsif.2012.0968

**Published:** 2013-04-06

**Authors:** Adam L. MacLean, Cristina Lo Celso, Michael P. H. Stumpf

**Affiliations:** 1 Theoretical Systems Biology, Division of Molecular Biosciences, Imperial College London, Sir Ernst Chain Building, London SW7 2AZ, UK; 2 Immunology and Infection, Division of Cell and Molecular Biology, Imperial College London, Sir Alexander Fleming Building, London SW7 2AZ, UK

**Keywords:** population biology, stem cells, haematopoiesis, approximate Bayesian computation, robustness

## Abstract

Haematopoietic stem cells (HSCs) are responsible for maintaining immune cells, red blood cells and platelets throughout life. HSCs must be located in their ecological niche (the bone marrow) to function correctly, that is, to regenerate themselves and their progeny; the latter eventually exit the bone marrow and enter circulation. We propose that cells with oncogenic potential—cancer/leukaemia stem cells (LSC)—and their progeny will also occupy this niche. Mathematical models, which describe the dynamics of HSCs, LSCs and their progeny allow investigation into the conditions necessary for defeating a malignant invasion of the niche. Two such models are developed and analysed here. To characterize their behaviour, we use an inferential framework that allows us to study regions in parameter space that give rise to desired behaviour together with an assessment of the robustness of the dynamics. Using this approach, we map out conditions under which HSCs can outcompete LSCs. In therapeutic applications, we clearly want to drive haematopoiesis into such regimes and the current analysis provide some guidance as to how we can identify new therapeutic targets. Our results suggest that maintaining a viable population of HSCs and their progenies in the niche may often already be nearly sufficient to eradicate LSCs from the system.

## 1. Introduction

Haematopoietic stem cells (HSCs) are somatic stem cells which reside in the bone marrow and produce all blood cell lineages [1]. The key characteristic of an HSC is its *stemness,* which is defined by the following criteria: it must have the ability to self renew, enabling maintenance of a stem cell pool; it must be able to generate multiple cell types through asymmetric division; and regenerate tissue when transplanted after cultivation *ex vivo*; finally, it should also be able to quiesce, enabling a long lifetime [2]. Resulting from their ability to respond to their environment in such a variety of ways, HSCs and the haematopoietic system are robust to deviations from the steady state, for example, following injury [3]. Despite the successful characterization of the process of haematopoiesis, there remain still large gaps in current understanding; for example, it is not known what mechanisms lie behind cell fate choices that lead to maintenance of the stem cell pool as well as the correct mix of differentiated myeloid and lymphoid cells, and how in detail HSCs interact with their environment in health and disease.

HSCs and their behaviour appear to be also characterized by their dependency on the so-called *niche*. In stem cell biology, the HSC niche comprises the complete set of environmental signals originating from HSC progeny, bone marrow stroma cells and other factors that regulate HSC function [4,5]. There has been much recent work on specifying the location of the HSC niche within the bone marrow [6–8] and identifying the cells and features that characterize the HSC niche. Whilst osteoblasts have been shown to play a crucial role in this niche [9,10], support grows for the roles of other cells as key niche factors [11].

Owing to the uncertainty about the anatomical constituents of the niche, an ecological approach is taken here. In ecological terms, a niche refers to the position of an organism within its environment defined by its habitat and the finite set of resources available to it. It is this broader definition that we will adopt throughout this paper. Our modelling approach is thus flexible enough to encompass unknown components because these are (implicitly) contained within the definition of an ecological niche as considered here. We will argue below that this ecological perspective allows us to capture essential aspects of HSC biology and, in particular, the interplay between normal HSC lineages and leukaemic stem cells and their progeny.

Leukaemia, a cancer of the blood, which can occur in all types of blood cell, originates in the bone marrow [12]. There are several proposed theories for the cellular composition of cancerous cell populations, the predominant of which is the cancer stem cell theory [13]. This states that within a population of cancer cells there exists a sub-population of cells which exhibit stem cell-like properties and which are primarily responsible for the maintenance of the cancer and its regeneration following therapy [14]. Thus, within a tumour population there exist cells analogous to the multipotent stem cells and lineage committed cells, which are also found in healthy tissue. While the exact identity of the cell of origin for different leukaemias is still unclear (and likely different), it is accepted that driving oncogenic translocations together with additional mutations are responsible for the transformation of normal haematopoietic cells into leukaemia stem cells (LSCs; [15]).

We are then faced with a situation where haematopoietic and leukaemia cells compete for the same niche and function in similar ways, responding to many of the same signals. Thus, it is natural to suggest that there exists direct competition for niche space and resources (such as interactions with niche-maintaining cells) between these species and their progeny. Studies have found evidence for such competition through the homing properties of LSCs [16] and in cases when HSCs are impaired either as a result of ageing or irradiation [17,18]. Although much attention has been paid to the molecular signalling and regulatory pathways controlling HSC behaviour and, to a lesser extent, how LSCs could interact with and disrupt this behaviour, to our knowledge there have been few studies that have framed this problem in terms of ecological competition (despite the fact that an increasing number of mathematical and computational approaches are gaining a foothold in stem cell biology [3,19–25]).

The basic mathematical analysis of ecological competition between multiple species is now well developed, and a host of analytical results have been obtained for the canonical models [26,27]. Here, we are most interested in the cell intrinsic factors that exert the greatest influence over the species they control, under the effects of competition from other species within the niche. There is a large body of work on the gene regulatory pathways which are involved in haematopoiesis and the related cancers [25,28–30]; we hope that by taking this slightly higher population-level view of the interacting species, we will be able to identify types of intervention that will allow us better to understand and control the differentiation and proliferation of competing HSCs and LSCs. The current perspective complements the molecular and mechanistic studies which are aimed at elucidating cellular decision-making processes. The population dynamics of cell lineages set constraints on the range of the dynamical behaviour of HSCs and their differentiation/proliferation behaviour and vice versa.

Whereas for some biological systems there is a wealth of data against which models can be fitted or compared with, in other cases, we have to base our analyses on less detailed information. Here, rather than estimating parameter values from what might be insufficient or irrelevant information, it is prudent to take a more general approach. With a few notable exceptions [31–34], we are not yet able to identify native HSCs *in vivo* with confidence and gather extensive amounts of data. It is possible to study leukaemia in mice, however, the experimental models available do not fully reflect the dynamics of leukaemia development taking place in patients. Xeno-transplantation of human leukaemia cells into immunocompromised mice is widely used, but it leads to inconsistent engraftment levels and does not take into account the role of the immune system in recognizing leukaemia development. Host conditioning prior to injection of human and mouse leukaemia cells is also widely used and provides a far from physiological starting point for the development of the disease. In experimental models that do not use conditioning, such as transgenic mice, leukaemia is initiated through expression of a driving oncogene in a specific cell population and not in a single leukaemia initiating clone through the use of artificial promoters. These initial conditions are very different to what we would expect to see in leukaemia patients where disease will typically arise from mutations in a single cell or lineage. Furthermore, recent results (both experimental and theoretical) suggest that within a population of cancerous cells, it may be hard to determine which proportion of cells exhibits stemness [35,36]. For these reasons, we proceed to explore large regions of parameter space and characterize the solutions they produce, rather than investigate models based upon any particular parameter set. By analysing models according to distinct outcomes that we select, we can test their robustness with regards to changes in input. We are thus able to home in onto dynamical regimes that exhibit certain types of behaviour in a computational affordable manner (even when other tools such as bifurcation analysis would be inappropriate because we are interested in quick responses). This, we propose, allows us to suggest new ways for therapies to coax the behaviour in the HSC niche into certain desirable directions.

## 2. Mechanistic models of the haematopoietic stem cell niche

### 2.1. Model assumptions

HSCs (species *S* in the model) are ancestors of all types of blood cells, a diverse set performing many different functions [4]. Here, we are not investigating all the possible blood cell fates, but the general mechanism of blood cell production; so we do not consider each type of blood cell individually, but group them all under the category of terminally differentiated blood cells (*D*). In between these and the stem cells, we group together all haematopoietic progenitor cells that are lineage committed and progressively lose self-renewal capacity (*A*). For the questions under investigation in this work, this coarse-grained version of the haematopoietic tree is sufficient [37]. All species are produced within the bone marrow ecological niche, but the population of differentiated cells, *D*, will leave the niche at a high rate as these cells enter the bloodstream; in healthy adult humans, for example, some two million new erythrocytes enter the bloodstream from the bone marrow every second [38].

Complementary to this model of the healthy haematopoietic system, we model leukaemia as two sub-populations of cancer cells, *L* and *T*. Here *T* are terminally differentiated leukaemia cells and *L* are proliferating leukaemia cells. We will mainly refer to the *L* population as LSCs, but in fact in our coarse grained model *L* represent any cell population driving the growth of leukaemia, including pre-LSCs, LSCs or leukaemia progenitor cells. This is important because both pre-LSCs and progenitor cells transforming to LSCs have been observed to play a role in the progression of leukaemias [16,39].

We do not have to model the intermediate stage in leukaemia differentiation explicitly (leukaemic species analogous to *A*). Species *A* is included because we are interested in how its dynamics change during disease progression; the effects of healthy species on a leukaemic progenitor population are of less interest. Furthermore, including an intermediate leukaemia species does not qualitatively change the results that are obtained. This statement is qualified in the electronic supplementary material, where a model with six species is analysed (see the electronic supplementary material, figure S1).

### 2.2. Niche competition

Competition models are based upon the ideas introduced by Lotka & Volterra and later ecologists, who consider two or more species that rely on the same limited (environmental) resource for their survival and proliferation [26,27,40]. In the case of haematopoiesis, this resource is the niche space available to stem cells, defined through the molecular signals necessary to maintain correct function [5].

In general terms, the competition, which exists between two species, *x* and *y*, where *X* and *Y* define the population size of each type, can be expressed in mathematical form as2.1
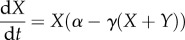
and2.2
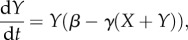
where *α* and *β* represent the growth rates of species *x* and *y*, respectively, *γ* is the strength of the feedback signal and *α*, *β*, *γ* ≥ 0; further examples of competition are given in Hofbauer & Sigmund [26]. This system assumes that the effect from feedback is linear and that both *x* and *y* experience feedback proportional to the combined population sizes of the two species. In a more general case, the feedback effect from (*X*+*Y*) could vary for each species, or the individual contributions could be altered. In this (simple) example, the equilibrium solutions can be trivially characterized. For all solutions with *α* ≠ *β*, the species with the greater growth rate will dominate and the other will go to zero. If (2.1) and (2.2) are generalized so that the feedback parameters for each term vary, coexistence or bistability (in which case the initial conditions affect the steady state taken up by the system) are also possible.

In the models presented here the number of species increases from two to five, but the number of parameters increases considerably as the model grows. In May [27], it is suggested that as species are added to a competition model, its stability, as measured by the smallest eigenvalue of the feedback coefficient matrix, will not increase but usually decreases. This general trend should be kept in mind when comparing real scenarios with the simple case described above.

### 2.3. Model A

**Figure 1.**
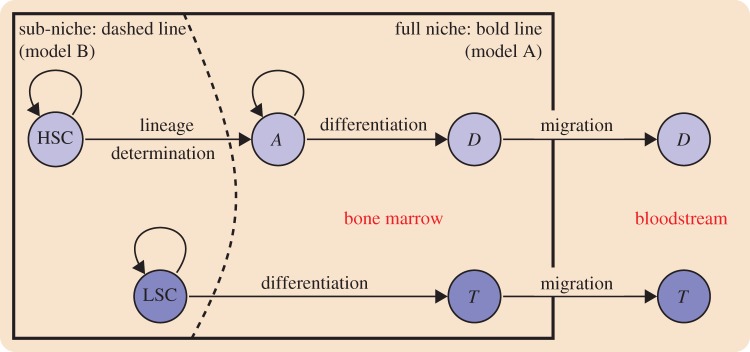
A model of competition within the HSC niche. Normal haematopoiesis occurs through HSC production of progenitor cells (*A*) that differentiate to mature blood cells (*D*). Alongside, a pool of mature leukaemia cells (*T*) is maintained by self-renewing LSCs. Mature cells are able to leave the niche by migration into the bloodstream.

In model A, we describe five species with competition modelled by linear feedback. The first three species are haematopoietic stem cells (*S*), progenitor blood cells (*A*) and terminally differentiated blood cells (*D*). Leukaemic stem cells (*L*) and fully differentiated leukaemia cells (*T*) complete the set (figure 1). The equations which fully specify the dynamics of model A are given below.2.3
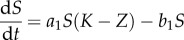
2.4
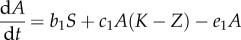
2.5
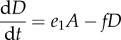
2.6
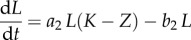
2.7
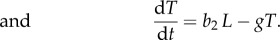

Here, *Z* = *S* + *A* + *D* + *L* + *T*, the parameter set (*a*_1_, *b*_1_, *c*_1_, *e*_1_, *f*, *a*_2_, *b*_2_, *g*) characterizes the phenotypes of the five species involved (table 1) and *K* represents the so-called *carrying capacity*, i.e. the total number of cells that can exist within the bone marrow. In this case *K* is ‘hard’ in the sense that it provides an exact upper limit for the total population size of the niche. (In model B below, the carrying capacity is ‘soft’ and is not equal to the maximal total population size.) In simulations *K* = 1 is used such that the population sizes are given as concentrations.

**Table 1.** Parameter set which describes model A.

parameter	definition
*a*_1_	rate of self-renewal of *S*
*b*_1_	rate of transition *S* → *A*
*c*_1_	rate of self-renewal of *A*
*e*_1_	rate of transition *A* → *D*
*f*	rate of disappearance of *D*
*a*_2_	rate of self-renewal of *L*
*b*_2_	rate of transition *L* → *T*
*g*	rate of disappearance of *T*

### 2.4. Model B

Model A appeals owing to its simple form that ought to facilitate explanation of its behaviour. There are, however, certain aspects of the haematopoietic system, which have not been included but which will be important biologically (and mathematically owing to the effects they would have on the dynamics of the system). These are addressed with our second model. Here, stem cell fates are modelled explicitly; that is, the terms controlling the rates of symmetric renewal (*S* → 2*S*), asymmetric renewal (*S* → *S* + *A*), symmetric differentiation (*S* → 2*A*) and loss of *S* (*S* → ∅︀) are incorporated explicitly (similarly for *L*). This is in contrast to, for example, *a*_1_ in model A which accounts for production of species *S* by any means. This allows for more detailed investigation of the relationships between fate choices, at the cost of increasing the size of the parameter space. In Mangel & Bonsall [37], haematopoietic stem cells and their descendants were modelled in a similar fashion. There feedback terms associated with stem cell fates were given an exponential form; in model B, this form is adopted. It is arguably more realistic that as the niche fills (or becomes crowded) the feedback effect increases faster than linearly, due, for example, to complex patterns of feedback from other cells in the niche.

The third important distinction between the models regards, therefore, the form of the niche. Within the bone marrow niche the location of HSCs (and LSCs) may be limited to specific sub-regions governed by particular signals [10,41]. In order to capture this behaviour, we separate the feedback terms into one containing the stem cell species *S* and *L*, and one containing all other species.

The assumption that there exists no overlap of species between these sub-niches may be oversimplifying matters in principle, but ought not to matter in practice as we will argue later. Thus, for the present at least, this assumption is upheld, also in an attempt to keep model complexity under control and maintain parsimony. Equations (2.8)–(2.12) describe the dynamics of model B, and equations (2.13)–(2.15) specify the feedback.2.8
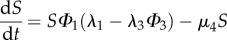
2.9
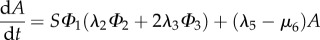
2.10
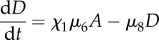
2.11
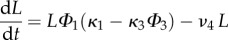
2.12
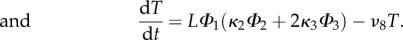


Where2.13
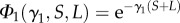
2.14
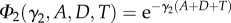
2.15
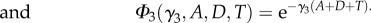


The parameter set required to fully specify the phenotypes of these species is now (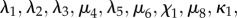 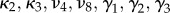). These quantities are described in Table 2. In our model analysis, the first 13 of these parameters are varied; for simplicity we assume that *γ*_1_, *γ*_2_ and *γ*_3_ that control feedback are identical and held fixed at the values *γ*_1_ = *γ*_2_ = *γ*_3_ = 0.01 (resulting in an arbitrary rescaling of other parameters). The feedback terms, equations (2.13)–(2.15), account for the effects of the occupants of each sub-niche on all differentiating species (*S*, *A* and *L*). *Φ*_1_ describes the effect of *S* and *L* on themselves; *Φ*_2_ and *Φ*_3_ describe the effects of *A*, *D* and *T* on the differentiating species.

**Table 2.** Parameter set which describes model B.

parameter	definition
*λ*_1_	rate of symmetric renewal of *S* (*S* → 2*S*)
*λ*_2_	rate of asymmetric renewal of *S* (*S* → *S* + *A*)
*λ*_3_	rate of symmetric differentiation of *S* (*S* → 2*A*)
*μ*_4_	rate of migration/apoptosis of *S*
*λ*_5_	rate of amplification of *A*
*μ*_6_	rate of disappearance of *A*
*χ*_1_	rate of transition of *A* → *D*
*μ*_8_	rate of migration/apoptosis of *D*
*κ*_1_	rate of symmetric renewal of *L* (*L* → 2*L*)
*κ*_2_	rate of asymmetric renewal of *L* (*L* → *L* + *T*)
*κ*_3_	rate of symmetric differentiation of *L* (*L* → 2*T*)
*ν*_4_	rate of migration/apoptosis of *L*
*ν*_8_	rate of migration/apoptosis of *T*
*γ*_1_, *γ*_2_, *γ*_3_	terms controlling feedback strength

The models presented here rely on certain other key assumptions which ought to be made explicit. The forms of *L* and *T* are similar to their healthy counterpart species: *S* for *L* and *A* or *D* for *T*. Model A assumes that only *D* or *T* can leave the niche by migration. This is not the case for model B, where all species can migrate or die. In model A, *T* has the same form as *D*; in model B, *T* is expressed as a combination of the structure of *A* and *D*. That is, *T* is produced directly from *L* but cannot self-replicate.

## 3. Robustness analysis via approximate Bayesian computation

The problem of classifying the steady state solutions of models of complex systems is often substantial. However the rewards of such efforts—a qualitative analysis of the results a model may provide—are great. Analytical expressions for the steady states can be obtained by solving the system of ordinary differential equations (ODEs) after setting each equation to 0. Analysis of these provides information about what equilibria can be reached and how stable they are [42]. However, here, we do not take an analytical approach but a numerical one. First, because the analytical results only hold in the limit *t* → ∞, whereas using our method we reach the steady states in finite time. Second, because the idea of any biological steady state in cancer other than extinction or metastasis is dubious—such states should be referred to as pseudo-steady states—taking an approximate approach to the analysis of the steady states in the model seems best.

By sampling parameter sets and simulating a model from these, we can obtain unbiased dynamical trajectories as examples of a model's behaviour that we can classify, according to some suitable criteria. Here, we classify trajectories according to whether HSC lineages win, LSC lineages win, or neither lineage wins (either due to coexistence, bistability or because the result falls into neither category). Obtaining enough samples for such outcome-clustering to be meaningful, however, may be difficult in practice, whenever the number of parameters affecting the system's dynamics exceeds five or six. We can use Latin hypercube (LH) or Sobol sampling approaches in order to sample more evenly over the entire parameter space, but even so coverage is often too sparse [43]. For a model with 10 parameters, for example, we require in excess of 1 000 000 samples to obtain just four samples per dimension. Furthermore, even given successful classification of a model's solutions, dissecting or further analysing such clustering within a high dimensional space is challenging. This is particularly true when we are investigating parameters that vary over orders of magnitude.

Rather than following such a global (but non-adaptive) sampling strategy, we can target our investigation by systematically identifying regions in parameter space where a model produces some desired behaviour. That is, rather than fitting a model to experimental data, we specify (surrogate) data that correspond to a system state that we are interested in. The conditions under which this type of behaviour is realized are initially unknown, but can be identified with approximate Bayesian computation (ABC). Originally developed for cases where computing the likelihood is not feasible, which is often the case for large, complex systems, ABC methods provide a convenient solution [44] to identify those parameters that have a high probability of having generated the data. ABC methods forego evaluation of the likelihood in favour of comparing real with simulated data [45,46]. If simulated data *x*′ for some parameter value *θ*′ is found to be in good numerical agreement with the observed data, *x*_0_, then *θ*′ is accepted as a valid draw from the (unknown) posterior distribution3.1
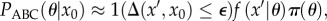
where *π*(*θ*) summarizes our prior knowledge/expectations on *θ* and 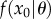 is the data-generating model, *Δ*(*a*,*b*) is a suitable distance function and 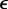 is our tolerance level, which determines how closely real and simulated data have to agree. ABC methods can also be used in the context of Bayesian model selection in order to choose the best model (given the data and compared with the alternative models considered; [47]). Bayesian model selection approaches strike an implicit balance between the predictive power of a model, its complexity (by favouring more parsimonious models over larger models unless these describe the data significantly better) and robustness. However, the same approach can also be used to scan models for the ability to generate certain outcomes. Here, we are interested in situations where there is competition between HSCs and LSCs, and in particular, we want to understand under what conditions the ‘ecological’ interactions between the two cell types (and, of course, their progeny) favours the HSCs and ideally results in suppression of LSCs.

**Figure 2.**
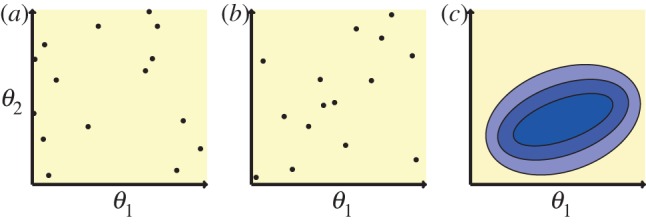
Three different ways to sample over the two-dimensional parameter space of *θ*_1_ and *θ*_2_. (*a*) Random sampling draws from a multivariate uniform distribution. This is the most general (undirected) approach. (*b*) By subdividing the space using a LH, more even sampling of the space is obtained. (*c*) In contrast to the global approaches of (*a*) and (*b*), ABC sampling is targeted towards regions of acceptable model behaviour, as represented by the blue circles growing darker with each iteration. the advantage of such an approach is that time is not wasted searching through those areas of space that we are not interested in.

To this end, we use qualitative inference to seek to determine, 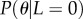, i.e. the probability distributions over parameters, *θ*, that—reliably and efficiently—yield loss of LSCs (and stable HSC pools). Our method is qualitative in the sense that we do not fit our model to surrogate data, instead we fit it to criteria that specify the behaviour we require: stable, positive populations of healthy cell species and vanishing populations of leukaemia species. The ABC sequential Monte Carlo (SMC) sampler of Toni *et al.* [44] allows us to do this and is already implemented in the ABC Sys-Bio package [48]. ABC SMC proceeds by approximating the *posterior* distribution, 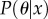 (where *x* is some data or specified system behaviour), in an iterative (sequential) fashion. Compared with other sampling methods of large-dimensional parameter spaces, e.g. random, LH and Sobol sampling [43], these Bayesian methods have the advantage of homing in onto regions in parameter space that have a high probability of resulting in the desired or observed outcome, and do not waste much time on exploring the generally vast regions of parameter space that do not lead to the specified outcome (figure 2). The sequential nature of the SMC framework also incorporates a natural adaptive/learning component, which here allows us to assay potential model behaviour efficiently and comprehensively [49].

## 4. Characteristics of desirable behaviour

**Figure 3.**
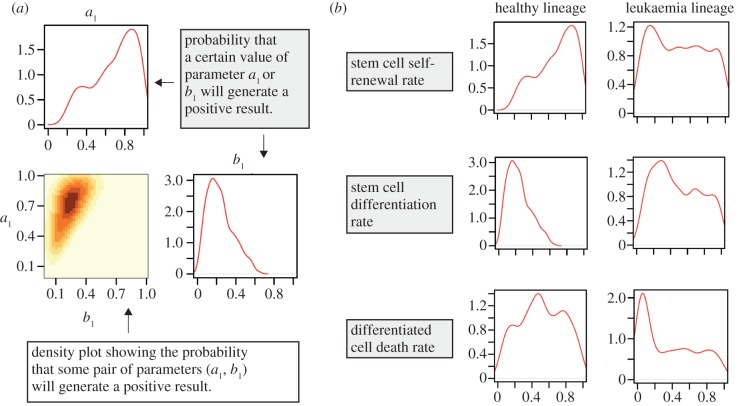
(*a*) An example of how the results are presented, with a description of their meaning. (*b*) Comparison of different rates for healthy and leukaemia cell species.

We investigate the circumstances under which the two models favour HSCs whilst suppressing LSCs, that is, *S*, *A* and *D* > 0 and *L* = *T* = 0 on reaching steady state. Each parameter was sampled from a uniform prior distribution in the range [0,1], since the parameters in the model are presented as rates. The posterior distribution that we obtain shows the regions of parameter space that are most likely to suppress leukaemia. Each parameter is described by a histogram; pairs of parameters can be visualized on two-dimensional density plots. This is illustrated in figure 3*a*.

Overall, we see that those parameters controlling LSC dynamics are less well inferred (less peaked) than those controlling HSC dynamics. This suggests that requiring a species to die out is a less stringent condition than requiring a species to reach some finite population size, as is the case for haematopoietic species *S*, *A* and *D*. A comparison between the parameters controlling healthy and leukaemia dynamics is shown in figure 3*b*. The full set of results for model A is given in the electronic supplementary material, figure S2.

The posteriors that we obtained appear to be unimodal. Therefore, principal component analysis (PCA) was performed on the posterior distribution for the parameters in order to evaluate the robustness of these systems to (joint) changes to parameters [50,51]. The principal components are constructed by finding the eigenvalues and eigenvectors of the covariance matrix of the parameters. The first principal component (corresponding to the largest eigenvalue) corresponds to the direction where the posterior is widest; the last principal component (corresponding to the smallest eigenvalue) points into the direction of least variance. This provides us with an indication of those parameters that are tightly constrained by the imposed/desired system behaviour; i.e. varying these parameters by even moderate or small amounts can have appreciable effects and differences in the system output (in the vernacular of Sethna and co-workers [52] these are the ‘stiff’ parameters). Here, these smallest principal components are of primary interest unlike in most cases, where PCA is used to try to identify parameters that control the largest variance in the data (i.e. distinguish between outcomes). Such ‘sloppy’ parameter (combinations) are less critical for determining system dynamics and may sometimes even not be identifiable (i.e. extend over the whole prior range).

For model A, the majority of the last principal component (56%) was composed of contributions from the parameters *a*_1_, *b*_1_ and *e*_1_. The second last principal component was mainly composed (63%) of *e*_1_ and *f*. So, in order to suppress leukaemia by populating the niche with healthy blood cells, it is most important to observe specific rates of the production of progenitor blood cells, self-renewal of the stem cells that are producing them, production of terminally differentiated cells from progenitor blood cells and of the migration/death of these differentiated cells.

**Figure 4.**
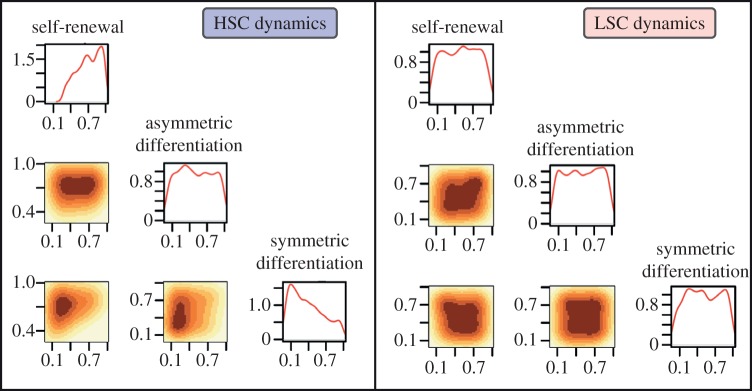
Dynamics of healthy and leukaemia stem cells that allow suppression of leukaemia to occur. For HSCs, self-renewal and symmetric differentiation should be controlled. For LSCs, no control is required. See figure 3*a* for explanation of the plots.

For model B, the 13 free parameters were also sampled from uniform priors on [0,1]. Parameters controlling healthy cell dynamics are better inferred than parameters controlling leukaemia. As in the case of model A, the constraint 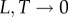 at equilibrium is more easily met than the constraint for HSC species to reach a finite positive value above some threshold. In figure 4, the posterior distribution for the stem cell dynamics is shown. The full output for model B is shown in the electronic supplementary material, figure S3.

We see in figure 4 that the posterior for HSC asymmetric differentiation is uninformative. The result that more information is given about self-renewal and symmetric differentiation rates suggests that controlling the rate of HSC asymmetric differentiation does not assist in achieving LSC suppression. Controlling self-renewal and symmetric differentiation of HSCs is sufficient in this case. In contrast to the parameters controlling HSC fate, none of the parameters controlling LSC fate need to be specified in order to achieve leukaemia suppression.

PCA was also performed on the results for model B. The last principal component is dominated (66%) by *λ*_1_, *λ*_3_ and *μ*_4_, which collectively control the fate of HSCs. This suggests that leukaemia can be beaten if a healthy population of HSCs is present. The second last principal component is mainly composed (64%) of *λ*_5_ and *μ*_6_. These parameters control healthy progenitor cell (*A*) dynamics and are analogous to *e*_1_ and *f* in model A. There is also a significant contribution (8%) from *ν*_4_ to this principal component, suggesting that of all the parameters controlling leukaemia, the death rate of LSCs could influence model dynamics the most.

## 5. Discussion

Here, we tried to determine under what circumstances leukaemia stem cells and their progeny succumb to normal HSCs and their progeny. We have taken a population-level perspective that models a highly simplified representation of the ‘ecology’ in the haematopoietic niche. In order to interpret these results, we have to address two questions: first, according to our simplified model, which parameters control the ecological balance, and which interventions would allow us to shift this balance in favour of normal non-leukaemic HSCs maintaining themselves and their progeny in the niche? And second, how can we relate these highly idealized model results to the processes occurring in the real world? The answer to the second question must depend on the answer to the first question and we address them in turn.

The results from both models suggest, perhaps somewhat counter-intuitively, that it is more important to maintain the dynamics of the HSC lineage within certain bounds than controlling parameters related to the LSC lineage. One reason for this is that there are many scenarios where suppression of the LSC lineage is relatively straightforward, but where nevertheless the HSC lineage cannot be maintained, recovers very slowly, or where both lineages coexist. This is perhaps the most important lesson emerging from both models.

The models presented here were developed based on the biological feature set we aim to describe: the crucial components of which are stem cell differentiation, competition for an ecological resource and feedback mediated by the niche. By characterizing solutions according to the lineage outcomes, we show that we can compare the likelihood of niche dominance by one lineage or another, given a certain model. There are certain regions of parameter space that produce results that are not permissible biologically, such as infinite population sizes. This is the case for model B in the region 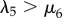, where results are unbounded. This can be deduced both from ABC parameter inference, which excludes this region even for large tolerances, and from simulation. So if species *A* is self-replicating more quickly than it is disappearing, the system is unstable.

The niche (of size *K*) can be divided into sub-niches, *K* = *K*_1_ + *K*_2_ as there is some evidence that HSCs and by extension LSCs reside in specific sub-compartments of the (ecological) niche. We must consider whether this coarse-grained description is realistic, or whether in reality we have a situation whereby some species move between sub-niches. Our central findings, however, appear to be robust features of niche-mediated feedback: in particular, we believe that maintenance of the HSC lineage is important to drive down LSCs and their progeny. In turn, an assault on LSCs and their progeny on its own will likely not suffice to restore the haematopoietic system.

There are many different ways to study the behaviour of a biological system. In this work, we look at a model globally, by performing targeted investigation of its whole behaviour space. This is facilitated by ABC. Thus, we can understand the stability and robustness properties of our system. In other studies [53], robustness is defined as the ability of a system to maintain its functions under perturbations, and contrasted with stability which has the narrower definition of maintaining certain system states. Using ABC, we can characterize not only a system's stability but also its robustness by looking at the maintenance of system functions, in this case the survival of the haematopoietic lineage.

Throughout the analysis of the models presented here, we have been considering the case where the presence of LSCs is already established. This should, we feel, include pre-clinical dynamics as well. Small leukaemia populations could occur often in the ecological niche and be outcompeted by healthy species thus restoring normal haematopoiesis. These instances are interesting because they provide more clues about the conditions favourable for suppressing an invasion of the niche by leukaemia cells.

Models must strike a balance between simplicity and reality. In the endeavour to retain or, more correctly, establish simplicity, many known aspects of the biology of haematopoiesis have been left out of the current analysis. For example, more complicated hierarchies and feedback from the periphery into the niche could be included. However, here too, details of the processes involved are largely unknown, and the explorative use of techniques such as ABC, which combine the ability to condition on desired or expected (quantitative and qualitative) behaviour with an assessment of the robustness of this behaviour are useful. In the future, such methods will aid experimental design and enable the study of how external (therapeutic) targets can be designed to control patterns of HSC differentiation.
